# Supplementary figures and images for: Microbial communities display alternative stable states in a fluctuating environment
Source: PLoS Comput Biol. 2020 May 26;16(5):e1007934. doi: 10.1371/journal.pcbi.1007934 (PMC7274482; doi:10.1371/journal.pcbi.1007934)

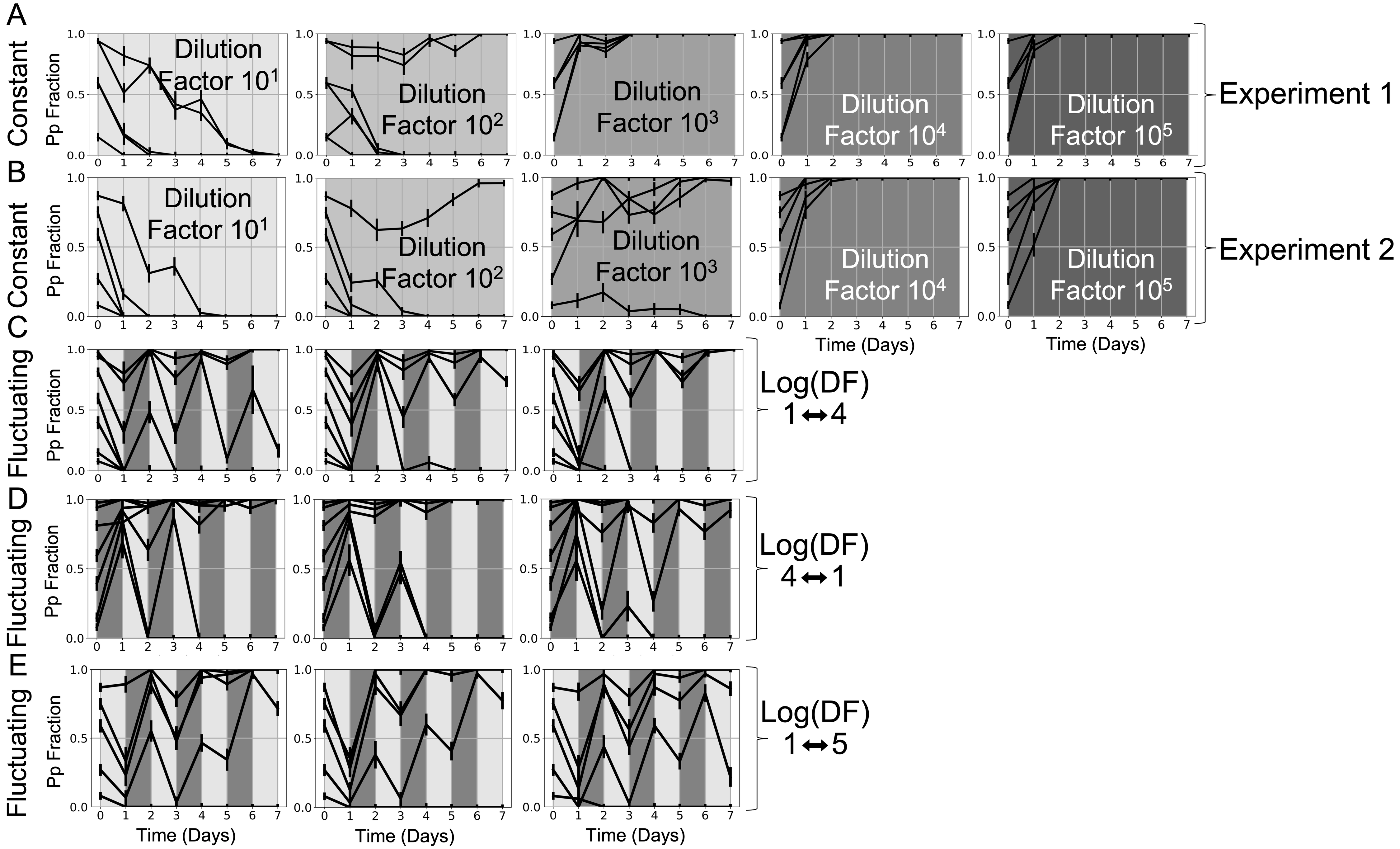

Supplement: S1 Fig — A-B: The data used to generate Fig 2E of the main text shows that Pp-Pv is a reproducibly bistable pair at dilution factor (DF) 102, and is sometimes bistable at DF 103, although the lowest starting fraction in Experiment 2 might have been above the separatrix. In both cases, the separatrix changes slightly between experiments. Slow-growing Pv is reproducibly dominant at DF 101, and fast grower Pp is dominant above DF 103. Two technical replicates (replicates of the experiment from the same colonies) of three starting fractions are shown in A, and a second biological replicate (replicates of the experiment from different colonies) of five starting fractions is shown in B. C-E: Alternative stables states form in a fluctuating environment, and the outcome trends toward that of the average DF (102.5 in panels C-D, 103 in panel E). Note that the starting fractions near the separatrix (~0.6–0.8 in panels C-D, ~0.1–0.3 in panel E) take the longest to equilibrate, and may need longer than seven days to reach an absorbing boundary. Each plot shows one technical replicate of seven starting fractions. Panels C and D show data sampled from the same biological replicate as panel A; panel E from the same biological replicate as panel B. Error bars are the SD of the beta distribution with Bayes' prior probability (see Methods). (TIF) [file pcbi.1007934.s001.tif]

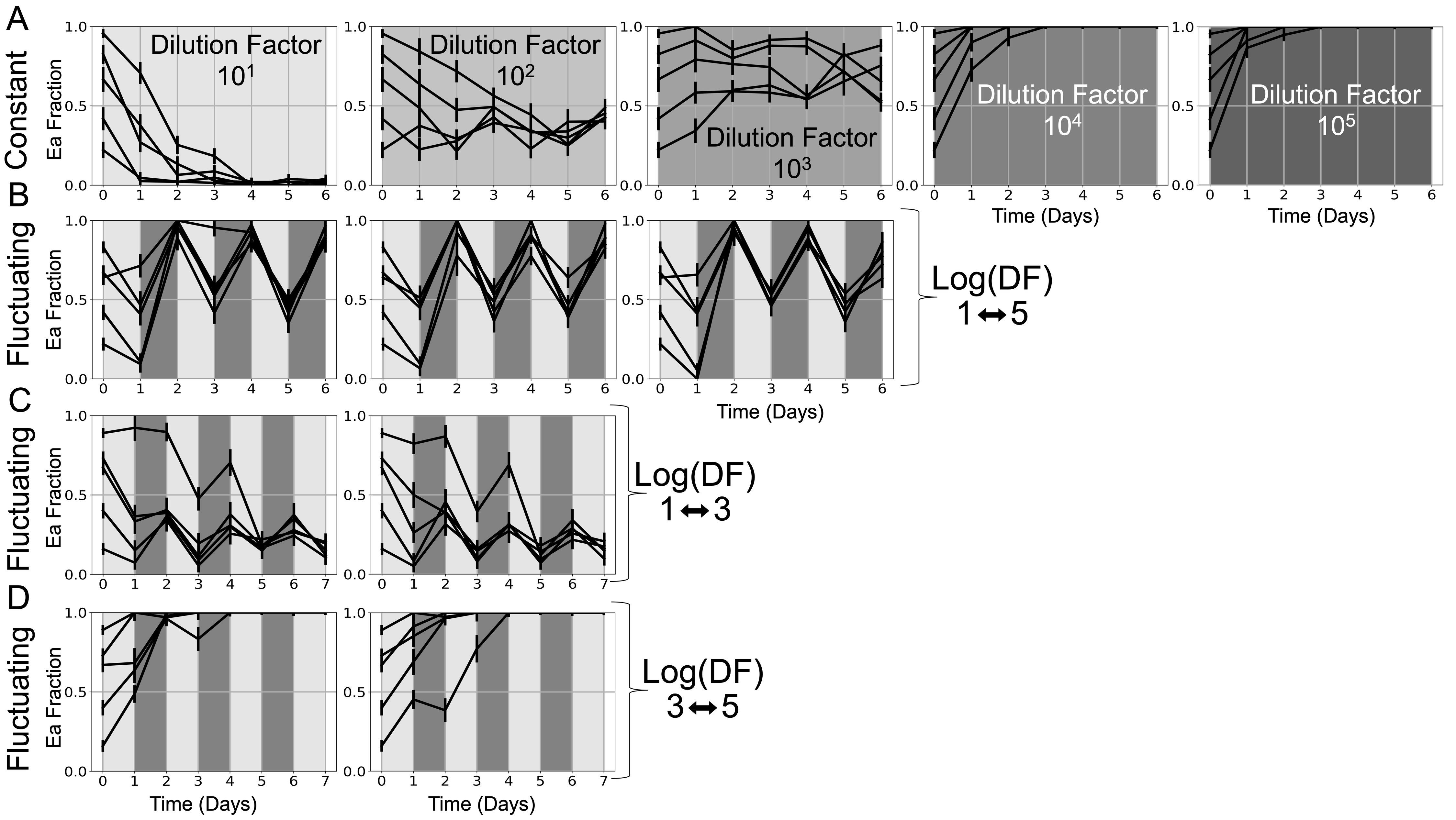

Supplement: S2 Fig — A: The data used to generate Fig 3D of the main text shows a reproducible shift as dilution factor (DF) increases, from dominance of slow grower Pv to coexistence to dominance of fast grower Ea. Each plot shows one technical replicate of five initial fractions. B-D: Coexistence results in a fluctuating environment if it also results in a constant environment subject to the average DF (103 in panel B, 102 in panel C). Furthermore, the coexisting fractions in the fluctuating environments match those of the constant environments. Additionally, competitive exclusion of Pv results in a fluctuating environment when it also results in the constant DF (104, panel E). Each plot shows one technical replicate of five initial fractions. Panels C and D were sampled from a different biological replicate than panels A and B. Error bars are the SD of the beta distribution with Bayes' prior probability (see Methods). (TIF) [file pcbi.1007934.s002.tif]

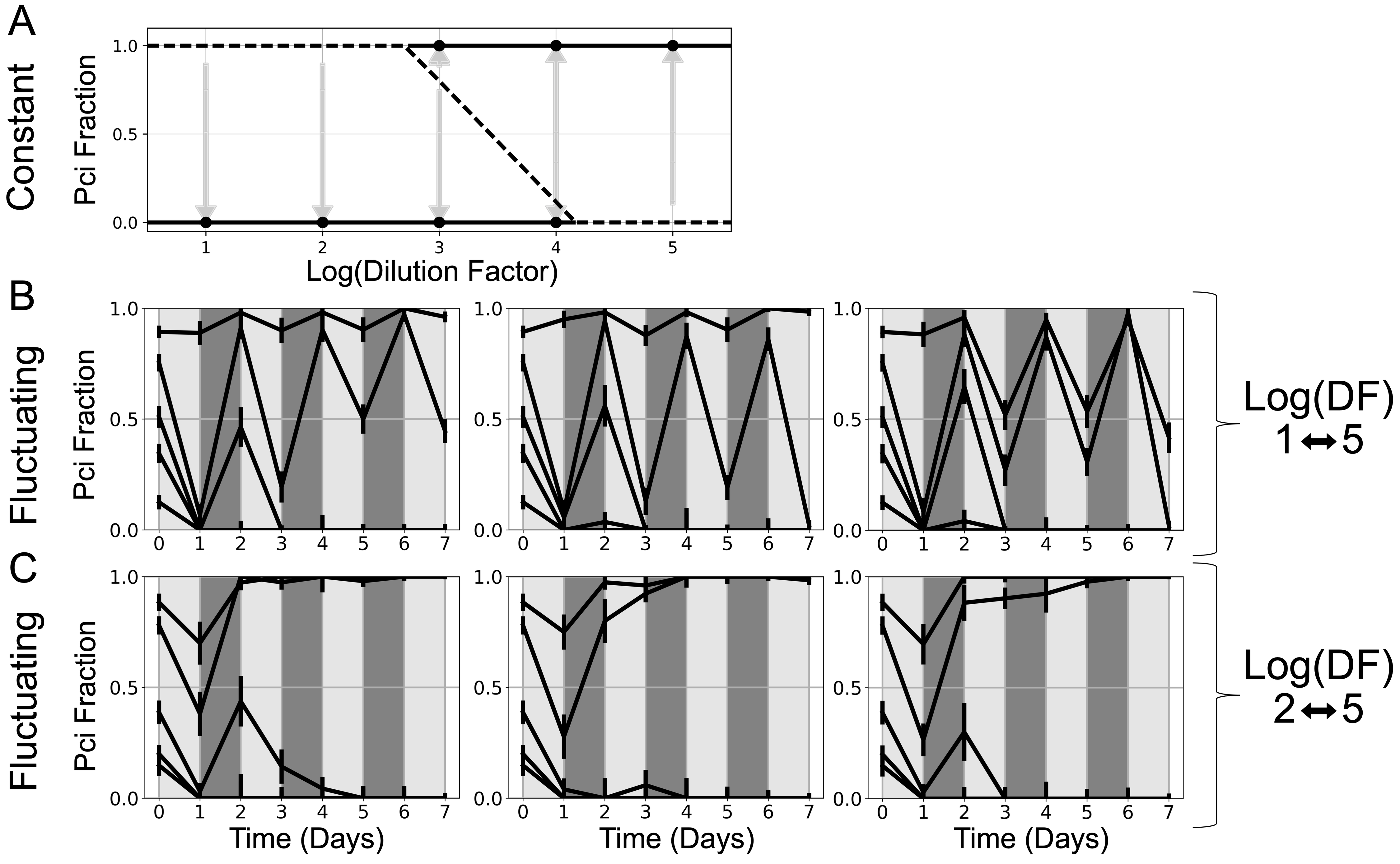

Supplement: S3 Fig — We conducted experiments with another pair found to exhibit alternative stable states, as can be seen from the bifurcation diagram in A, which includes data from two different biological replicates. B: In an environment fluctuating between DF 101 and DF 105, most trajectories reach the absorbing boundary of zero; the highest initial fraction of Pci is very close to the separatrix in the equivalent constant environment (DF 103) and as such does not consistently go to a single final outcome. One should note that these results do not violate the time-averaging prediction of the LV model, since perturbations near a separatrix may cause a trajectory to cross the separatrix and thus take longer to reach equilibrium. Each plot shows one technical replicate of five initial fractions. C: The outcome is more predictable when we fluctuate between DF 102 and 105 for a constant equivalent environment of DF 103.5, in which the estimated separatrix is about midway between the absorbing boundaries of one and zero. Here we see alternative stable states forming in the fluctuating environment more clearly, depending on whether a starting fraction is closer to one or zero. Each plot shows one technical replicate of five initial fractions, all of which were drawn from a different biological replicate than in panel B. Error bars are the SD of the beta distribution with Bayes' prior probability (see Methods). (TIF) [file pcbi.1007934.s003.tif]

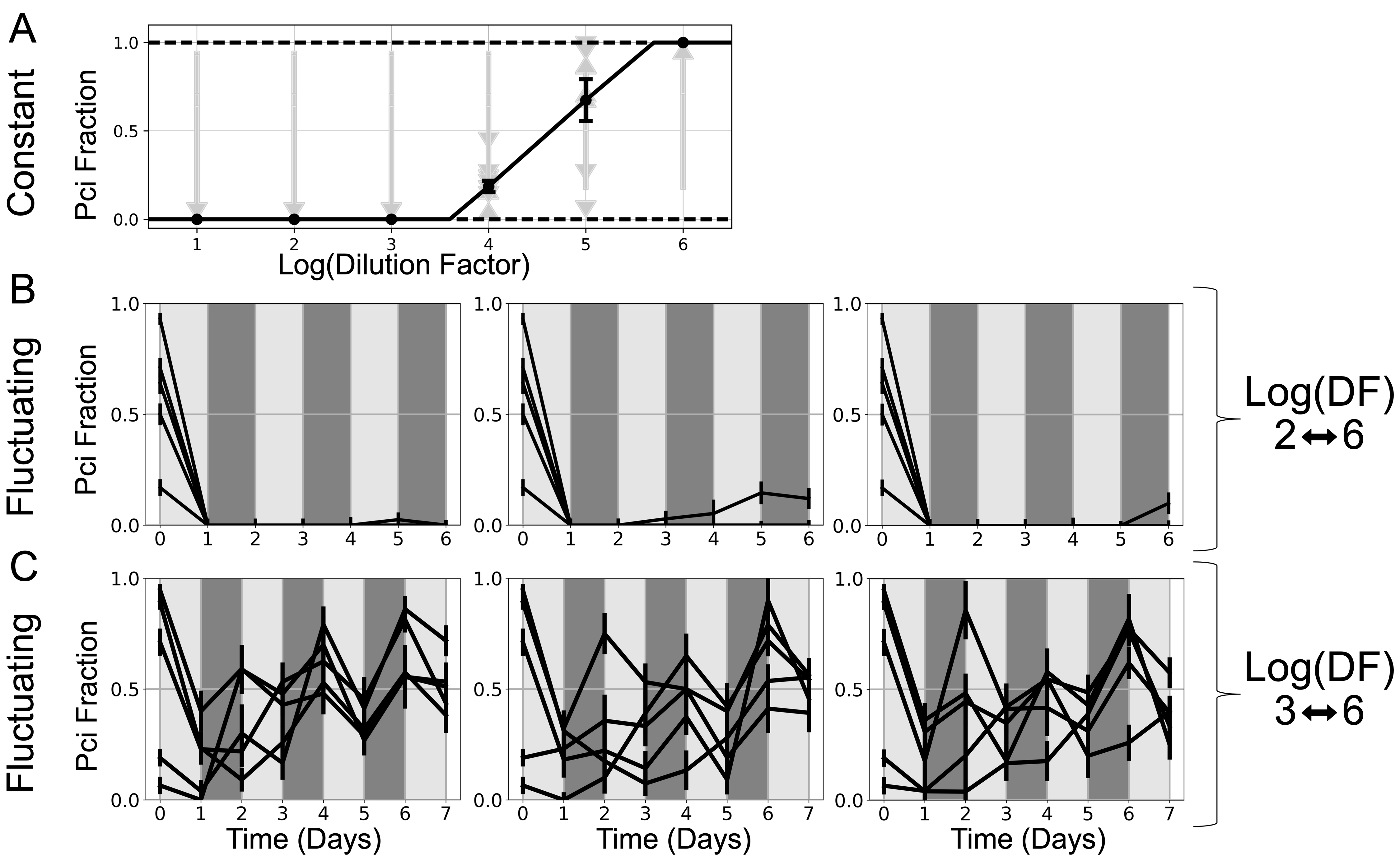

Supplement: S4 Fig — We conducted experiments with another pair found to coexist, as can be seen from the bifurcation diagram in A, which includes data from two different biological replicates. Error bars are the SEM of all replicates (n = 6; 2 biological replicates of 3 starting fractions each). B: In an environment fluctuating between DF 102 and DF 106, for a constant equivalent environment at DF 104, the model predicts coexistence at a stable fraction of Pci of ~0.2, as seen in A. The failure of coexistence here may be due to stochastic extinction, or domination by Pa in the first 24-hour cycle. Neither of these occurrences would violate the time-averaging prediction of the model. Each plot shows one technical replicate of five initial fractions. C: In an environment fluctuating between DF 103 and 106 for a constant equivalent environment of 104.5, we expect a stable coexisting fraction further from exclusion. As such, we more clearly see the coexistence of the species in this fluctuating environment. Each plot shows one technical replicate of five initial fractions, all of which were drawn from a different biological replicate than in panel B. Error bars are the SD of the beta distribution with Bayes' prior probability (see Methods). (TIF) [file pcbi.1007934.s004.tif]

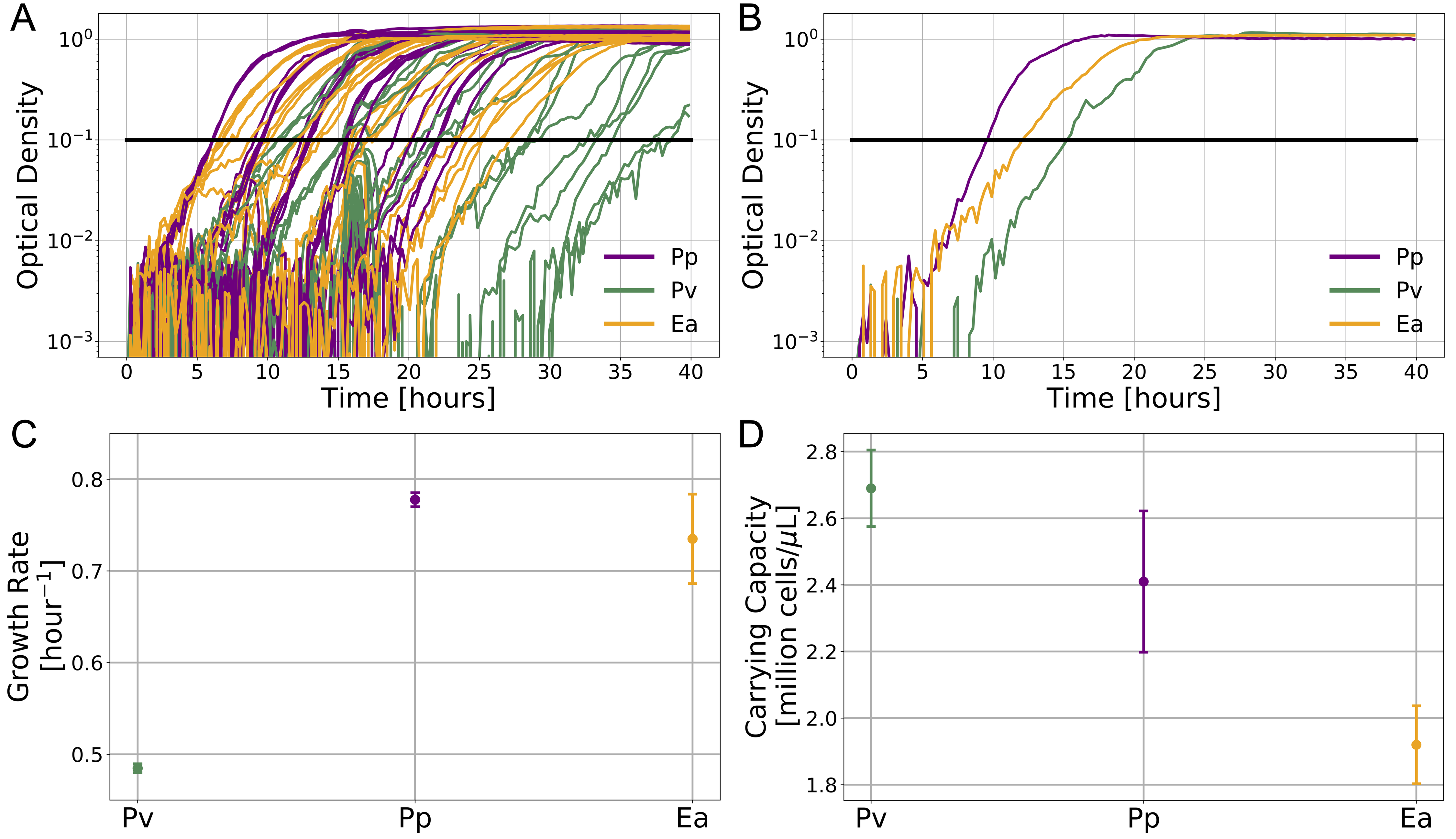

Supplement: S5 Fig — To measure growth rates, species were grown in monoculture from a low starting density, with optical density (OD) measured over a period of ~40 hours. Before these measurements, species were grown in 1X LB broth overnight, and then transferred to the experimental medium for 24 hours. The OD of all species was then equalized. The resulting cultures were diluted into fresh medium at factors of 10−7 to 10−3. All of the data used to determine the growth rates is shown in A, and one set of growth curves is shown in B. Background noise has been subtracted from all curves, and no curves have been smoothed. A threshold OD of 0.1 was chosen, and exponential growth was assumed to occur until this threshold. The time each monoculture took to reach this threshold OD was used along with its initial OD to determine the growth rate. By assuming exponential growth to a threshold, we assume no lag time occurs, but the resulting measurement implicitly incorporates lag: longer lag times will cause the measured growth rate to be lower, while shorter lags will have the opposite effect. C: Final growth rate measurements were determined for each species by averaging these measurements across all replicates. D: Shown are the measured carrying capacities used for simulating the LV model shown in the figures in the main text. Error bars are the SEM of all replicates (n≥16, per species), of which there were four biological replicates and at least four technical replicates of each (plate reader noise due to factors such as condensation caused some replicates to be excluded for some species). (TIF) [file pcbi.1007934.s005.tif]

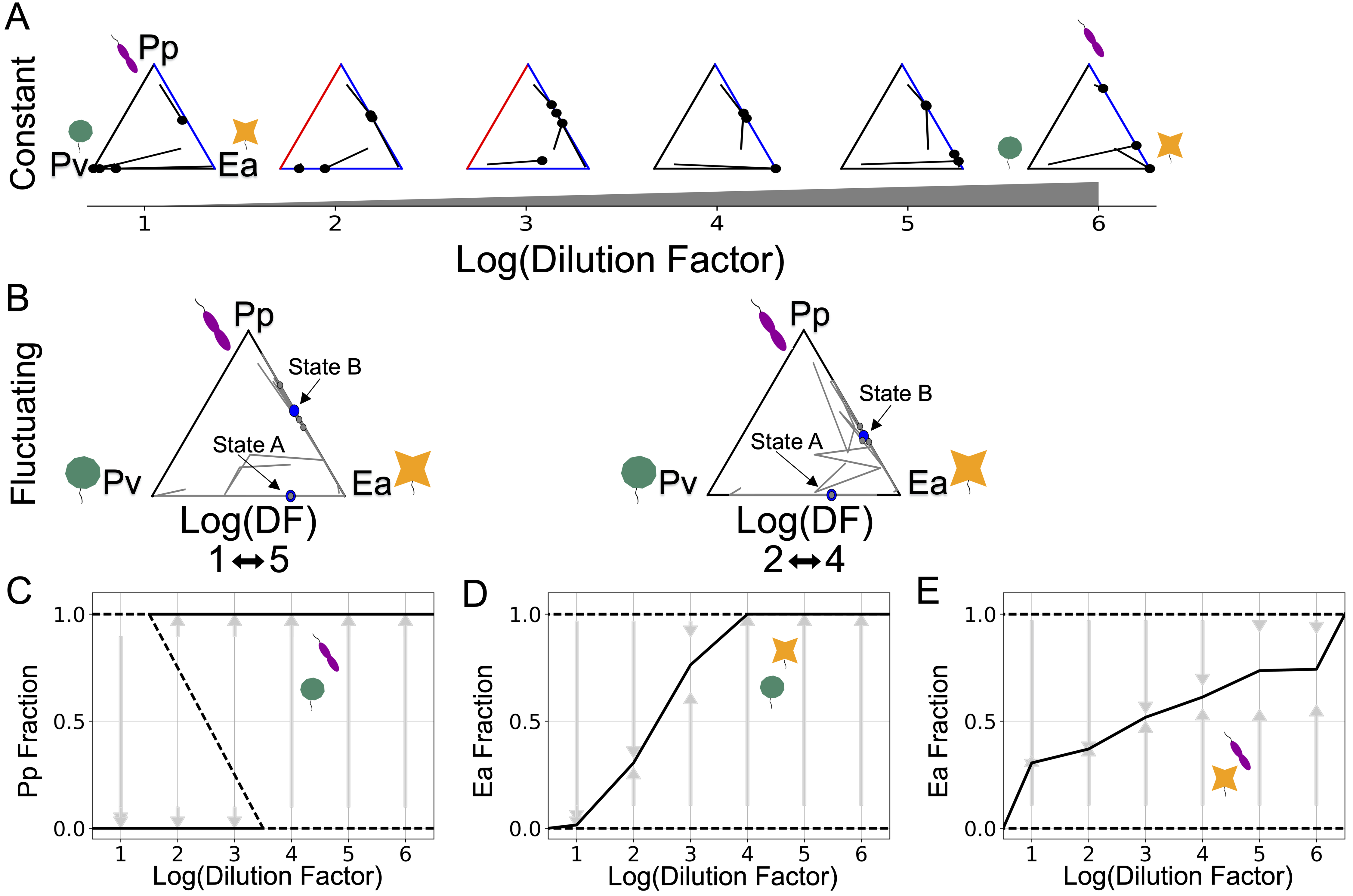

Supplement: S6 Fig — A: Initial and final fractions for each of four starting conditions in each of six constant environments, in which the daily dilution factor (DF) ranged from DF 101 to DF 106. The edges of the ternary plots denote pairwise outcomes; black indicates exclusion, blue coexistence, and red bistability. These outcomes were determined by competing each pair of species in constant environments, the results of which can be seen in the bifurcation diagrams of C-E. (Note that one of the pairs, Ea-Pp, violates the model’s prediction: although Pp is the faster grower (see S5 Fig), Ea is slightly favored at higher dilution factors. However, Ea and Pp had the most similar estimated growth rates (p = 0.17, compared to p < 0.01 for the other two pairs of species), which makes the model’s prediction more tenuous for this pair.) In B, the results of fluctuating the DF in two different regimes are shown, between DF 101 and DF 105, and between DF 102 and DF 104. Both have a constant equivalent DF of 103, and we see that both regimes have the same qualitative outcomes as the constant environment with DF 103, as three initial fractions go to State B and one initial fraction goes to State A, with state labels consistent with Fig 4 in the main text. All plots show data sampled from the same biological replicates. (TIF) [file pcbi.1007934.s006.tif]
