# Supplementary material for: Microbial communities display alternative stable states in a fluctuating environment
Source: PLoS Comput Biol. 2020 May 26;16(5):e1007934. doi: 10.1371/journal.pcbi.1007934 (PMC7274482; doi:10.1371/journal.pcbi.1007934)
Supplement: S1 Text — (DOCX) [file pcbi.1007934.s007.docx]

**S1 Text: Derivation of Lotka-Volterra model modified by added death**

The most basic form of the two-species Lotka-Volterra model takes the following form:

$$\begin{aligned} \frac{\dot{N_{i}}}{N_{i}}= r_{i}-c_{ii}N_{i}-\sum_{j} c_{ij}N_{j}\#\left( 1 \right) \end{aligned}$$

where $r_{i}$ is the exponential growth rate of species $i$ (minus any intrinsic death rate), $c_{ii}$ is the rate at which species $i$ inhibits itself, and $c_{ij}$ is the rate at which species $j$ inhibits species $i$. Equation (1) can be re-parameterized to:

$$\begin{aligned} \frac{\dot{N_{i}}}{N_{i}}= r_{i}\left( 1-\frac{N_{i}-\sum_{j} \beta_{ij}N_{j}}{K_{i}} \right)\#\left( 2 \right) \end{aligned}$$

where $K_{i} =\frac{r_{i}}{c_{ii}}$ is the carrying capacity and $\beta_{ij} =\frac{c_{ij}}{cii}$ is the competition coefficient. We can further re-parameterize the model by normalizing by carrying capacity:

$$\begin{aligned} \frac{\dot{\hat{N_{i}}}}{\hat{N_{i}}}= r_{i}\left( 1-\hat{N_{i}}-\sum_{j} \alpha_{ij} \hat{N_{j}} \right)\#\left( 3 \right) \end{aligned}$$
where $\hat{N_{i}}=\frac{N_{i}}{K_{i}}$ and $\alpha_{ij}=\beta_{ij}(\frac{K_{j}}{K_{i}})$. This version of the model is useful because the competition outcomes depend upon whether the competition coefficients are greater or less than one: stable coexistence occurs when both coefficients are less than one, bistability when both are greater than one, and dominance/exclusion when only one coefficient is greater than one. This leads to the log/log phase space (Fig 2B, Fig 3A, S7 Fig), in which boundaries form where competition coefficients equal one.

The modified Lotka-Volterra model includes an added global death term:

**Supplementary Figure 7:** Re-parameterization of model allows for division of phase space where competition coefficients equal one.

$$\begin{aligned} \frac{\dot{\hat{N_{i}}}}{\hat{N_{i}}}= r_{i}\left( 1-\hat{N_{i}}-\sum_{j} \alpha_{ij} \hat{N_{j}} \right)-\delta\#\left( 4 \right) \end{aligned}$$

This term can be absorbed in order to return the model to its previous form (Equation (3)):

$$\begin{aligned} \frac{\dot{\tilde{N}_{i}}}{\tilde{N}_{i}}= \tilde{r}_{i}\left( 1-\tilde{N}_{i}-\sum_{j} \tilde{\alpha}_{ij}\tilde{N}_{j} \right) \#\left( 5 \right) \end{aligned}$$

where $\tilde{r}_{i}=r_{i}-\delta,$ $\tilde{N}_{i}$= $\frac{\hat{N_{i}}}{1-\frac{\delta}{r_{i}}}$ and $\tilde{\alpha}_{ij}=\alpha_{ij}\frac{1-\frac{\delta}{r_{j}}}{1-\frac{\delta}{r_{i}}}$. Multiplying $\alpha_{ij}$ by a term means that we add a term to ${log \alpha}_{ij}$. Due to symmetry, the same term will be subtracted from $\log\alpha_{ji}$. As a result, increasing death causes the outcome to move in a line with a slope of negative one through the log/log phase space (S7 Fig), beginning at the outcome with no added death, (${log \alpha}_{ij}$, ${log \alpha}_{ji}$). If this outcome resides in the quadrant where the slow grower wins, increasing death will eventually result in the fast grower winning. If the trajectory begins in the quadrant where the fast grower wins, however, increasing death will not change the outcome.

While a global death rate of $\delta$ leads to the simple prediction that the fast grower is favored, it is not the most realistic scenario. In reality, different species may be affected by different added death rates. In this case, the expression for the competition coefficients becomes:

$$\begin{aligned} \tilde{\alpha}_{ij}=\alpha_{ij}\frac{1-\frac{\delta_{j}}{r_{j}}}{1-\frac{\delta_{i}}{r_{i}}} \#\left( 6 \right) \end{aligned}$$

Taking the log of Equation (6) results in addition of a term to ${log \alpha}_{ij}$, the same term which will be subtracted from ${log \alpha}_{ji}$. The outcomes will therefore still move along the same 45° line through the phase space, although they will not necessarily move in the direction that favors the faster grower. Added mortality will favor the faster grower if the following condition is met:

$$\begin{aligned} \frac{\delta_{s}}{\delta_{f}}>\frac{r_{s}}{r_{f}} \#\left( 7 \right) \end{aligned}$$

We therefore see that the fast grower can still be favored if it is killed at a higher rate (as in the case of $\beta$-lactam antibiotics, which target faster growers by inhibiting cell wall biosynthesis). Furthermore, the growth/competition tradeoff at low dilution is not required to observe outcome changes if the slow grower is selectively targeted; in this case, the trajectory would move from fast grower winning at low mortality, to coexistence or bistability at intermediate mortality, to the slow grower winning at high mortality.
