# Supplementary material for: Microbial communities display alternative stable states in a fluctuating environment
Source: PLoS Comput Biol. 2020 May 26;16(5):e1007934. doi: 10.1371/journal.pcbi.1007934 (PMC7274482; doi:10.1371/journal.pcbi.1007934)
Supplement: S2 Text — (DOCX) [file pcbi.1007934.s008.docx]

**S2 Text: Dilution factors can be time-averaged in the Lotka-Volterra model**

As noted in the main text, the Lotka-Volterra model makes an interesting prediction about a fluctuating mortality rate. Because the per-capita growth rates $\frac{\dot{N}_{i}}{N_{i}}$ are linear and additive, a fluctuating mortality rate can be time-averaged for the purpose of finding the equilibrium steady state. In fact, even a discrete mortality process, such as the daily dilution factors that we use in experiments, can be time-averaged. The outcome of alternating daily dilution factors (for example, 10^1^ and 10^5^) is the same as the outcome of a constant dilution factor equal to the geometric mean of the two (10^3^).

To show that the equilibrium state resulting from different dilution factors is equivalent to that from the time-averaged dilution factor, we will begin by examining a single dilution factor. In contrast to a continuous dilution rate, such as in a chemostat, the daily dilution process is itself a fluctuating mortality rate. During most of the cycle, the mortality rate is zero, and the dilution process can be thought of as an instantaneous spike (delta function) in mortality. We will assume that a culture is allowed to grow for a time of length $T$ and then diluted in an instant, whereby the number of cells is divided by the dilution factor $DF$. We can begin by time-averaging the Lotka-Volterra model to model the process:

$$\begin{aligned} \frac{1}{T}\int_{0}^{T} \frac{\dot{N}_{i}}{N_{i}}dt=\frac{1}{T}\int_{0}^{T} \frac{d}{dt}\log\left( N_{i} \right)dt=\frac{1}{T}\int_{0}^{T} r_{i}\left( 1-N_{i}-\alpha_{ij}N_{j} \right)dt \#\left( 1 \right) \end{aligned}$$

Additionally, we will assume that enough cycles have passed such that the system has already reached equilibrium by time $t=0$. In this case, $N_{i}$ grows to the same quantity each cycle, $N_{i}\left( T \right)$, before being diluted to the same quantity, $N_{i}\left( 0 \right)$. The ratio of the two quantities is equal to the dilution factor:

$$\begin{aligned} \frac{1}{T}\int_{0}^{T} \frac{d}{dt}\log\left( N_{i} \right)dt=\frac{1}{T}\log\left( \frac{N_{i}\left( T \right)}{N_{i}\left( 0 \right)} \right)=\frac{\log\left( DF \right)}{T}\#\left( 2 \right) \end{aligned}$$

Now plugging the right-hand side of equation (2) into the left-hand side of equation (1) and defining the time-average $\frac{1}{T}\int_{0}^{T} x dt=\left\langle x \right\rangle$, we can re-write the time-averaged LV model:

$$\begin{aligned} \frac{\log\left( DF \right)}{T} =\frac{1}{T}\int_{0}^{T} r_{i}\left( 1-N_{i}-\alpha_{ij}N_{j} \right)dt\#\left( 3 \right) \end{aligned}$$

$$\begin{aligned} r_{i}\left( 1-\left\langle N_{i} \right\rangle-\alpha_{ij}\left\langle N_{j} \right\rangle\right)-\frac{\log\left( DF \right)}{T}=0 \#\left( 4 \right) \end{aligned}$$

We removed $r_{i}$ and $\alpha_{ij}$from the integrals because they are constants. Equation (4) tells us that at equilibrium (where the per-capita growth rate is equal to zero), the dilution process is equivalent to subtracting a continuous death rate equal to $\frac{\log(DF)}{T}$. A daily dilution will therefore lead to time-averaged population densities that are the same as a continuous dilution rate of this magnitude.

Now that we have shown that, at equilibrium, a daily dilution is equivalent to a continuous dilution rate, we can proceed to show that, at equilibrium, an alternating daily dilution factor is equivalent to a constant time-averaged dilution factor. We will assume that a culture is allowed to grow for a time of length $T$, then diluted by factor $DF_{1}$, then grown for another time of length $T$, and then diluted by factor $DF_{2}$:

$$\begin{aligned} \frac{1}{T}\int_{0}^{T} \frac{\dot{N}_{i}}{N_{i}}dt=\frac{\log\left( DF_{1} \right)}{T}; \frac{1}{T}\int_{T}^{2T} \frac{\dot{N}_{i}}{N_{i}}dt=\frac{\log\left( DF_{2} \right)}{T}\#\left( 5 \right) \end{aligned}$$

Time-averaging the entire two-cycle process, we find (dividing each component by $2$ to account for the two cycles):

$$\begin{aligned} \frac{r_{i}}{2}\left( 1-\left\langle N_{i} \right\rangle_{1}-\alpha_{ij}\left\langle N_{j} \right\rangle_{1} \right)-\frac{\log\left( DF_{1} \right)}{2T}+\frac{r_{i}}{2}\left( 1-\left\langle N_{i} \right\rangle_{2}-\alpha_{ij}\left\langle N_{j} \right\rangle_{2} \right)-\frac{\log\left( DF_{2} \right)}{2T}=0\#\left( 6 \right) \end{aligned}$$

Simplifying Equation (6) reveals that the alternating dilution factor process is equivalent to one with a constant dilution factor equal to the geometric mean of the two:

$$\begin{aligned} r_{i}\left( 1-\frac{\left\langle N_{i} \right\rangle_{1}+\left\langle N_{i} \right\rangle_{2}}{2}-\frac{\alpha_{ij}\left( \left\langle N_{j} \right\rangle_{1}+\left\langle N_{j} \right\rangle_{2} \right)}{2} \right)-\frac{1}{T}\log\sqrt{DF_{1}DF_{2}}=0\#\left( 7 \right) \end{aligned}$$

To see this equivalence, remember that the constant dilution factor process is modeled with equation (4). Equation (7) can be mapped to equation (4) by redefining the following parameters:

$$\begin{aligned} DF= \sqrt{DF_{1}DF_{2}}; \left\langle N \right\rangle=\frac{\left\langle N \right\rangle_{1}+\left\langle N \right\rangle_{2}}{2}\#\left( 8 \right) \end{aligned}$$

A daily dilution regime that fluctuates between dilution factors $DF_{1}$ and $DF_{2}$ is thus equivalent, at equilibrium, to one with the same dilution factor everyday equal to the geometric mean of the two factors, $\sqrt{DF_{1}DF_{2}}$.

If the fluctuating environment leads to coexistence of two species, we expect the constant environment to also lead to coexistence of those two species. The same is true of bistability. However, in a finite system, if the system spends too much time in one dilution factor, the outcome may differ from the deterministic solution. For example, suppose species A wins in $DF_{1}$, species B wins in $DF_{2}$, and both species coexist in $\sqrt{DF_{1}DF_{2}}$. In a real system, it takes a finite amount of time for species B to go extinct in $DF_{1}$. If the first cycle is longer than that amount of time, the outcome of the fluctuating environment will be that species A wins rather than coexistence.

The situation becomes even more precarious in the case of bistability: too much time in one dilution factor may move the system to the other side of the separatrix. We thus expect that the location of the separatrix could change when going from a constant dilution factor experiment to an alternating dilution factor experiment, or even that bistability might not result in both regimes. Remarkably, in our experiments, we found that the separatrix was approximately the same in both types of experiments. If we had used an alternation scheme lasting longer than one cycle (for example, two cycles at $DF_{1}$ followed by two cycles at $DF_{2}$), it is unlikely that this would still be true.
