# Supplementary material for: Microbial communities display alternative stable states in a fluctuating environment
Source: PLoS Comput Biol. 2020 May 26;16(5):e1007934. doi: 10.1371/journal.pcbi.1007934 (PMC7274482; doi:10.1371/journal.pcbi.1007934)
Supplement: Attachment — Submitted filename: 3-22_Review_response.docx [file pcbi.1007934.s009.docx]

We would like to thank the reviewers for carefully reading our manuscript and providing helpful feedback. We believe that their feedback helped to clarify our results and to provide more context about their importance to future readers. Below, we provide responses to the reviewers’ comments and show how the manuscript has changed.

(Note: Line numbers refer to the clean document, rather than the document with tracked changes.)

Reviewer #1: Abreu et al. present an interesting suite of experiments and modeling results, which demonstrate how a fluctuating (density-independent) mortality rate is equivalent to an equivalent time-averaged rate for determining whether a small set of soil-derived bacterial species coexist in co-culture. The experimental methods are straight-forward, as is the LV modeling framework, and I have no technical issues with the manuscript. Overall, I think this is a valuable contribution to the literature. I have a few points that the authors may want to consider in the discussion & interpretation of their results, outlined below.

Specific Comments:

1) In your title, you play up 'alternative stable states', but your model also predicts coexistence (i.e. if the alphas are both negative). Maybe state 'coexistence or alternative stable states'?

We agree that it should be clear that coexistence can also result in the fluctuating environment, and we included this in the abstract. We did not include it in the title, because coexistence in a fluctuating environment has been discussed exhaustively in the literature, and has perhaps led some to take this outcome for granted. This is why we motivated this study by showing that alternative stable states can emerge in a fluctuating environment in Figure 1.

2) While your linear model does apply to these 3 strains under the range of fluctuation conditions applied, it is likely that you will find parameters that push your system outside this linear range. Did you try to push your system beyond this range? For example, as you state in the text, large enough variation between dilution rates will result in bottlenecks at the higher magnitude dilutions, which in turn will lead to stochastic extinction events.

In most cases, we explicitly aimed to avoid stochastic extinction. We did see one example of what looks to be stochastic extinction, though, in a pair shown in Supplementary Figure 4B (copied here, see below). We expected coexistence at a low fraction when averaging to dilution factor 10^4^ (alternating between 10^2^ and 10^6^), but most replicates reached the absorbing boundary of extinction. On the other hand, we observed expected coexistence when averaging to dilution factor 10^4.5^ (alternating between 10^3^ and 10^6^), because the expected coexisting fraction is close to 0.5 at that dilution factor:

We have added the following sentence (in **bold**) to the Discussion (Lines 259-260):

“We used regularly alternating and moderate disturbances, but as the duration and intensity of disturbances increases one expects that eventually the model’s predictions will fail due to stochastic extinction caused by finite population sizes. **(See S4B Fig. for an example of apparent stochastic extinction in most replicates, when coexistence at a low fraction was expected.)**”

3) From a coexistence/diversity perspective, I think you can include some discussion of a spatial interpretation of your alternative stable states as being a form of coexistence. For example, if you have spatial heterogeneity (i.e. spatial patches) then isolated communities that end up in either stable state will show a mixture of species at the 'landscape' scale (i.e. across patches). Lots of good ecology work showing how this type of patch-heterogeneity can promote diversity/coexistence, & prevent extinctions at the landscape scale (but usually in the context of dispersal and neutral patch dynamics -- bistability adds a cool new twist).

We agree that this is a great suggestion, and have mentioned this point in the Introduction (new sentence in **bold**, lines 69-71):

“While empirical observations of fluctuation-induced coexistence are common, less attention is focused on how perturbations may lead to outcomes other than increased diversity. For example, alternative stable states have been observed in fluctuating environments, such as in gut microbiota communities subject to laxative treatments^26^, intertidal biofilms perturbed by climatic events^27^, and regions of forest and barrens disturbed by frequent fires^28^. In these cases, however, it is often difficult to distinguish between alternative states that are simultaneously stable and different states that are stable in different environments. There is some theoretical support for fluctuation-induced alternative stable states in particular systems^29,30^, but these predictions are challenging to test in the field due to the difficulty of controlling all ecological factors. **Moreover, alternative stable states might appear to be coexistence in a spatially structured environment if isolated patches are occupied by different states; indeed, numerous studies have investigated the hypothesis that environmental heterogeneity increases diversity^31,32^.** The proposition that environmental fluctuations may have more complicated effects on ecosystems than simply affecting diversity is therefore in need of systematic study and demonstration.”

4) It's interesting that the pair of species that coexist at intermediate dilution rates are more phylogenetically dissimilar. May be worth discussion how the two Pseudomonads likely overlap more in their niches, compared with E. aerogenes? Indeed, Ea is the coexistence partner for both Pv and Pp in the 3-species co-cultures (i.e. alternate stable states include either Pv + Ea or Pp + Ea). Seems to fit with Ea having compatible niche requirements that mitigate competition? But this might also just be a coincidence.

We agree that it is worth mentioning that Pseudomonads usually prefer citrate over glucose, while Ea prefers glucose, and that our medium has both. This does not necessarily explain why Pp + Ea and Pv + Ea coexist, however. Coexistence on a single carbon source is often observed in the lab, and is even possible in a Monod resource model with discrete (not continuous) dilutions. Additionally, there is a coexisting pair of Pseudomonads in S4 Fig. The following discussion has been added to the Results (lines 189-195):

“This bistability of coexisting states can be predicted from the corresponding pairwise results, using our previously developed community assembly rules^35^. **(It is also worth noting the fact that both coexisting states, *Pp-Ea* and *Pv-Ea*, consist of a member of the *Pseudomonas* genus along with a member of the *Enterobacteriaceae* family. The former prefer to consume organic acids over glucose^36^, while the latter prefer glucose^36,37^. Our medium contains both glucose and citric acid, and this compatibility of niche requirements may mitigate competition. We also observed coexistence of two *Pseudomonas* species (S4 Fig), however, which demonstrates that this simple form of niche compatibility is not required for coexistence.)**”

5) Lines 253-256 -- It might be a bit strong to state that these prior studies 'lack clearly defined predictions and thus make less clear conclusions.' While I agree that your experimental system is quite simple/tractable and the modeling predictions are a bit more clear/cogent, many of these studies do include controlled perturbations in the lab coupled with LV modeling + predictions. I think there are some similarities with these prior studies that further support and complement your conclusions, which could be better leveraged to support your results.

For example, Rodrıguez-Verdugo et al. showed that increasing the frequency of environmental fluctuation resulted in the 2-species community behaving similar to a time-averaged environment, while very low frequencies of fluctuation produced very different results (i.e. one species doesn't have enough time to recover and goes extinct before the environment can flip back to the other condition; Figs. 2-3). This results seems to match quite well with your discussion in the last paragraph if the supplemental text, where you state that "too much time in one dilution factor may move the system to the other side of the separatrix."

Furthermore, in Gibbons et al. the authors show that different intensities and frequencies of disturbance, which ultimately add up to the same time-averaged disturbance rate, have equivalent effects on community diversity (see Fig. 5). In this study, intensity and frequency of two disturbance types (dilution and UV exposure) were modulated independently across (but not within) replicates, unlike your study where the frequency is kept constant for the same community while you modulate dilution intensity within community replicates. In either case, it didn't matter how 'disturbances' are delivered in time, as long as the community is exposed to the same average level of disturbance. Thus, it appears your time-averaging result may be applicable to more complex communities as well.

We agree that this is a better way to discuss these studies. We have re-worded that part of the Discussion as follows (lines 224-233):

“Although our results rely on what some may call simplistic predictions, our conclusions are strengthened by the simplicity of both the experimental system and the model. Furthermore, there is some evidence that averaging environmental disturbances may be a feasible strategy in more complex scenarios as well. In one study of multispecies microbial microcosms^39^, the multiplicative effect of intensity and frequency of disturbances determined the resulting diversity of a community, indicating that the average overall disturbance was more important than its timing. Other studies^15,40^ have found disturbance timing to be the key determinant of diversity within a community, although they did not compare timing to average disturbance. More investigation is needed to determine whether time-averaging of disturbances is useful in complex yet experimentally tractable communities.”

More g	enerally, we have re-framed the Discussion to emphasize that more work is needed to determine when time-averaging is feasible, rather than to emphasize smaller differences between our study and others.

Finally, I'd like to bring your attention to another paper not cited here by Turkarslan et al. (<https://www.embopress.org/doi/full/10.15252/msb.20167058>). As you discuss in your manuscript, dilution rate does not appear to influence the alphas (in the LV) or the maximal growth rates. However, other types of disturbances will impact the coefficients in such a model. One example is any fluctuation that forces task-switching (e.g. biphasic growth). Task-switching exacts a metabolic cost in organisms capable of growing under different metabolic regimes. Turkarslan et al. show how higher frequencies can lead to ecological collapse, when organisms are spending all their resources on task-switching without being allowed enough time to grow under either condition (i.e. fluctuation faster than lag phase). Interestingly, they show that if you delete the organisms' ability to sense the environmental change, they stop trying to switch between metabolic modes and collapse is averted. Just one example for how fluctuations that change the niche-landscape of an environment are qualitatively different from fluctuations that do not (e.g. density-independent mortality). Another relevant example would be how dilution/mixing frequency alters oxygen concentrations and therefore changes competition between colony morphotypes of P. fluorescens (e.g. <https://onlinelibrary.wiley.com/doi/full/10.1111/j.1558-5646.2009.00758.x>). Some discussion of this would be interesting to include, especially in the paragraph where you pose the question about 'which types of disturbances can be time-averaged[?]'.

We thank the reviewer for these suggested citations. We agree that a discussion of the effects of high-frequency fluctuations would add interesting context, particularly after we mention that low-frequency fluctuations could lead to stochastic extinction in our system. We have cited Turkarslan et al and another paper in the Discussion (lines 256-264):

“We used regularly alternating and moderate disturbances, but as the duration and intensity of disturbances increases one expects that eventually the model’s predictions will fail due to stochastic extinction caused by finite population sizes. (See S4B Fig. for an example of apparent stochastic extinction, in most replicates, when coexistence at a low fraction was expected.) **High-frequency fluctuations, on the other hand, may cause qualitatively different dynamics in systems where microbes are able to sense environmental changes^44,45^.**”

Reviewer #2: The authors set to test one of the main hypotheses in ecology, namely that environmental fluctuations promote species diversity. They conducted a set of experiments for a simple microbial community and predicted their outcomes with a Lotka-Volterra model with mortality. The authors demonstrated that the outcome of the community dynamics in the environment with a fluctuating dilution rate is the same as in the environment with a time-averaged mortality rate. Moreover, they showed that in different cases fluctuations might lead not only to coexistence but also bistability between two species. In my opinion, the paper outlines a great strategy on how to combine theoretical models and observable experimental data.

I have three main questions, which I think it would be helpful if the authors address when revising their manuscript:
1) It is unclear from the manuscript why the authors observed different outcomes in intermediate dilution rates for two pairs: fast grower Pp and slow grower Pv (bistability), fast grower Ea and slow grower Pv (coexistence). Is it due to the difference in interactions between these two pairs? On lines 140-142, while describing the model, the authors mentioned that “coexistence and bistability result when both alpha_ij are less than or greater than one”, but how can one distinguish between these two? Is there some qualitative understanding of these two cases? I think that it might be a useful discussion as according to the authors these are two different cases - one has increased diversity, another is not.

Unfortunately, we do not know the molecular mechanism of interaction between our species that leads to bistability (in some other cases, e.g. Ratzke and Gore, *PLOS Biology* (2018), we have found that bistability is driven by antagonistic changes to the pH, but this is not the mechanism in this pair). However, we would note that part of the power of the phenomenological approach taken here is that we can make accurate predictions regarding the outcome in the fluctuating environment even without knowledge of the molecular mechanism. Moreover, the modeling approach and predictions highlight the potential generality of the result. Nonetheless, we do know something about the carbon source preferences of the families of bacteria that we are using, and we have added the following lines to the Results (new sentence in **bold**, lines 189-195):

“This bistability of coexisting states can be predicted from the corresponding pairwise results, using our previously developed community assembly rules^35^. (**It is also worth noting the fact that both coexisting states, *Pp-Ea* and *Pv-Ea*, consist of a member of the *Pseudomonas* genus along with a member of the *Enterobacteriaceae* family. The former prefer to consume organic acids over glucose^36^, while the latter prefer glucose^36,37^. Our medium contains both glucose and citric acid, and this compatibility of niche requirements may mitigate competition. We also observed coexistence of two *Pseudomonas* species (S4 Fig), however, which demonstrates that this simple form of niche compatibility is not required for coexistence.**)”

2) I appreciate that the authors tried to demonstrate that their “time averaging” result holds even for a more complex community of tree species. However, I feel like this example is handpicked, from an earlier Friedman et al. 2017 study, where the authors found that there are no higher-order interactions for Ea-Pp-Pv trio and the outcome of their co-culture can be predicted well using pairwise interaction experiments. I wonder what will happen if there are some non-linear interactions between species (e.g. Pch-Pf-Pv trio from Friedman et al. 2017)? Could a Lotka-Volterra model with some quadratic terms and mortality still be time-averaged? Is there any way to generalize this result to the less specific case?

We thank the reviewer for reading our lab’s past work. However, our data is not drawn from Friedman et al, *Nature Ecology and Evolution* (2017). We used a different growth medium and a different dilution protocol than was used in that study (24hr vs. 48hr and by a different dilution amount). The only shared component is the species used, which have been stored in our lab freezer since Celiker et al, *Nature Communications* (2014).

To address your questions, it is actually true that linear pairwise interactions can lead to trio outcomes that violate Friedman’s assembly rules. It is also possible, of course, that nonlinear interactions could lead to trio outcomes, and in that case, time-averaging is not expected to be accurate. We have added a line mentioning this in the last paragraph of the Discussion (lines 272-273):

“A linear time-dependence of interactions and growth rates on added mortality may be more dominant in simple communities, while higher-order effects may become more important in complex communities. **If there are nonlinear higher-order interactions in a multispecies community, time-averaging may fail.** Additionally, our simple community results do not mean that there were no nonlinearities or covariances in our system, but only that they were not sufficiently large so as to alter the experimental outcome.”

We would very much like to know what would happen in a fluctuating experiment with one of Friedman’s three-species communities that violates the assembly rules, but we feel such experiments are beyond the scope of this project.

3) I am a bit confused about the authors mention that “models, such as those that explicitly define resource consumption with the Monod equation … do not make this prediction”. Could the authors elaborate further on why this is the case? Is there an easy way to generalize the result to any other consumer-resource model?

In the Monod equation, the per-capita growth rate is a non-linear Monod function:

$$\frac{\dot{N}}{N}=r\left( \frac{c}{K+c} \right)-\delta$$

Where $r$ represents the maximum growth rate, $c$ represents resource concentration, and $K$ is the Monod constant, the concentration of resources which results in the half-maximum growth rate.

You could employ a linear resource model with a linear per-capita growth rate, and in that model, the outcome of a fluctuating dilution rate can be time-averaged:

$$\frac{\dot{N}}{N}=rc-\delta$$

We have added the following text to the Discussion (lines 237-243):

“In the LV model, mortality/dilution $\delta$ can be time-averaged because it does not covary with other quantities, and the per-capita growth rates are linear and additive^25,34^. **Other ecological competition models, such as those that explicitly define resource consumption with the Monod equation, do not predict that the outcome of a fluctuating mortality rate would be the same as that of the equivalent constant environment, because of the nonlinearity of the Monod function with respect to resource concentration^13,41^. A resource-explicit model in which per-capita growth increases linearly with resource concentration^42^, on the other hand, would allow for mortality to be time-averaged. While such a model is unrealistic at high resource concentrations, it might represent low-concentration dynamics well.**”

Below are some of my minor concerns on the manuscript:
1) line 30 “cycles of of light”.

Fixed this typo.

2) I suggest not to abbreviate Lotka-Volterra in the manuscript or to do so at the first appearance (line 82).

We added the abbreviation at the first mention (line 86).

3) I suggest to introduce Pv as a “slow grower” and Pp as a “fast-grower” somewhere in the text (around line 100-104) as now these definitions appear in the caption of Fig 1.

We incorporated this suggestion and referred to S5 Fig, which shows the species’ growth rates in monoculture (lines 102-104):

“We began by coculturing **slow-growing** *Pseudomonas veronii* (*Pv*) and **fast-growing** *Pseudomonas putida* (*Pp*). **(These species’ growth rates were measured in monoculture to determine which grew faster (S5 Fig.).)**”

4) I suggest to highlight lines corresponding to outcomes (Pp or Pv wins) in the same color (e.g. purple for panel B and green for panel A) as species on top in Fig 1 and 2. Then it might be easier to distinguish different trajectories in panel C in case of intersecting lines.

We tried this suggestion but decided that it might confuse some readers, and because we couldn’t use such notation in Fig. 3 (the coexisting pair), it would be inconsistent to have different notation in Figs. 1 and 2.

5) I am a bit confused about double grey arrows in Fig 2E and Fig 3D for the intermediate dilution rates. Do they represent different replicates?

We have edited the caption for Figure 2E: “At each dilution factor, gray arrows represent time trajectories from initial to final fractions.”

6) I would recommend to create a separate legend for “shading” of dilution rates and add it in Fig 2,3,4. Also I think, it might be fine if light grey shade is added on top of Fig 4B to show similar pattern as on previous plots.

We have superimposed transparent gray shading on panel B—see picture below next comment.

7) I feel that it might be beneficial for the reader if authors somehow mark 4 starting points from Fig 4B (initial fractions of bacteria) in Fig 4A. Numbers or different colors probably would work.

We have numbered the starting points in panel A and added numbers to panel B. We agree this notation is beneficial:

8) The authors has demonstrated that for Ea-Pp-Pv trio their modified Lotka-Volterra model is capable of prediction the resulting state. Then, it should be possible to predict a “separator” between 2 attractor states on ternary plot in Fig 4A. Could it be shown on the plot?

As noted in the Supporting Information (lines 123-127), it is possible (and maybe probable) that the location of a separatrix could change in a fluctuating environment. Even assuming that the pairwise separatrices do not shift in the fluctuating environment, it is possible for the three-species separatrix to shift. Assuming it did not, in order to calculate the three-species separatrix, we would need to quantitatively fit the model to find estimates for interaction coefficients in the three pairs. We have found, through our work in both Abreu et al, *Nature Communications* (2019) and Friedman et al, *Nature Ecology & Evolution* (2017) that quantitatively fitting the Lotka-Volterra model does not generate accurate predictions (this is one reason why Friedman’s assembly rules were created). Instead, we believe that the qualitative predictions from this simple phenomenological model are valuable for understanding laboratory results. In this case, the qualitative prediction is that bistability results in both constant and fluctuating environments.

9) In Supplementary materials, the authors provided results of dilution experiments for two other pairs of species from the original Friedman et al. 2017 study. However, they are not mentioned anywhere throughout this manuscript and are not needed for the understanding of the main message of the paper. Is there a reason for keeping these?

These results were not taken from Friedman et al, *Nature Ecology & Evolution* (2017). They are recent results, and they provide additional evidence that the time-averaging prediction can be generalized to more species and pairs. We have forgotten to refer to them in the main text and appreciate the reminder. The following reference has been added to the Results (lines 173-176):

“In both the bistable pair (Fig. 2C-D) and the coexisting pair (Fig. 3B-C), simulations match the experimental trajectories over time, in both constant and fluctuating dilution factors. **To confirm that these results could be generalized to more species and pairs, we implemented time-averaging in two additional pairs, one bistable and one coexisting, as well as in other dilution factors with our primary pairs (see S1-S4 Figs.).** These examples of agreement between the model and experiments emphasize that in our simple bacterial communities, a fluctuating dilution factor may be time-averaged.”
